# Reply to McNally's comment on “Bioactive glass S53P4 vs. autologous bone graft for filling defects in patients with chronic osteomyelitis and infected non-unions – a single center experience” by Steinhausen et al. (2021)

**DOI:** 10.5194/jbji-6-203-2021

**Published:** 2021-05-27

**Authors:** Eva Steinhausen, Rolf Lefering, Martin Glombitza, Nikolaus Brinkmann, Carsten Vogel, Bastian Mester, Marcel Dudda

**Affiliations:** 1 Department of Orthopedic and Trauma Surgery, BG Klinikum Duisburg, University of Duisburg-Essen, 47249 Duisburg, Germany; 2 Institute for Research in Operative Medicine (IFOM), University of Witten/Herdecke, Cologne, Germany; 3 Department of Trauma, Hand and Reconstructive Surgery, University Hospital Essen, University of Duisburg Essen, Essen, Germany

We read Martin McNally's Letter-to-the-Editor (McNally, 2021) with great interest and are pleased to make precise the presentation of our results.

First, we will describe the differences between the patient collectives of the *Zeitschrift für Orthopädie und Unfallchirurgie* (*ZOU*) paper (Malat et al., 2018) and the current *Journal of Bone and Joint Infection* (*JBJI*) paper (Steinhausen et al., 2021).

The inclusion criteria, the enrolment period and the follow-up period of the studies were different.

Fifty patients were treated in the *ZOU* paper. Of these, 46 patients were treated with bioactive glass (BAG) and 4 patients with BAG in combination with autologous bone graft.

Ninety-five patients were treated in the *JBJI* paper. Of these, 51 patients were treated with BAG, 12 patients with BAG in combination with autologous bone graft and 32 patients with autologous bone graft only.

Thirty of 50 patients (*ZOU* paper) were excluded for the *JBJI* paper because they did not meet the additional inclusion criterion “follow-up of at least 12 months” in the *JBJI* paper. Therefore, 20 single individuals are published in both the *JBJI* and *ZOU* papers.

In these 20 patients, the follow-up data of 14 patients differ in the two publications. The patients in the *JBJI* paper have a longer follow-up due to the 18-month longer follow-up period (until January 2018 instead of June 2016). Six patients did not show up for further follow-up.

The first study (*ZOU*) is to be regarded as a study with preliminary results. All patients in whom we implanted bioactive glass up to February 2016 were analyzed, even if they only had a follow-up of 2 months. This is also listed as limitations in the *ZOU* paper.

We assumed the two studies to be independent of each other, with a new data set in the *JBJI* paper: preliminary results in the *ZOU* paper, longer follow-up in the *JBJI* paper, no control group in the *ZOU* paper. Therefore, we presented the results of the *ZOU* study in Table 4 in the *JBJI* paper. However, we agree that the *ZOU* paper should have been referenced in the methods section and in Table 4, given the connection between the two publications.

In order to be able to compare complication rates, the patient collectives must be comparable on the one hand, and the definitions must match on the other hand. A well-known problem of the diagnosis “osteomyelitis” is the extreme heterogeneity. Reinfection rates of 0 % up to 32.8 % are reported in the literature, depending on the definition and the treatment algorithm (Lam et al., 2019; Bose et al., 2015).

In the *JBJI* study, there is a higher number of patients with complex and therapy-refractory courses compared to the *ZOU* paper. In particular, patients of the *JBJI* study had a longer – unsuccessful – course of disease with an average of 33.8 months from accident to defect filling with bioactive glass, while patients of the *ZOU* study had an average of 21.5 months.

There may be a negative bias due to our retrospective study design: patients without complications or reinfection may not have visited during our consultation hours any more. Thus, patients without reinfection have more frequently a follow-up of < 12 months and therefore could not be included in the current *JBJI* study.

In addition, the definition of reinfection or persistence of infection used by other authors is less strict than ours. Other authors (Romano et al., 2014; Lindfors et al., 2017) define a “fair” outcome as a wound with prolonged drainage or serum leakage of up to 6 weeks. These findings would be interpreted as persistent infection/reinfection in our assessment.

The differences in definition as well as in the patient collective may explain the increased complication and reinfection rate in the *JBJI* study. Overall, the comparability of our two studies is limited, just like the comparability of complication rates with other studies.

As Martin McNally and we state, there are no large randomized trials directly comparing the use of bioactive glass. In our opinion, results of animal or in vitro studies – even if they are of high quality – are not sufficient to compare with the results of in vivo studies.

However, there are studies that prove the antibacterial effect of BAG without addition of local antibiotics. Van Vugt et al. (2016) evaluated the use of various bone graft substitutes in a systematic review. No significant differences were found between the different bone graft substitutes (with and without adjunctive use of local antibiotics).

According to our standard at that time, we used the synergy of the local antimicrobial effect of bioactive glass and systemic antibiotics according to the antibiotic resistance pattern to achieve optimal efficacy. A change in pathogens was seen in only 2 % of our BAG patients.

Independent of the effectiveness, some authors describe the tolerability of BAG as excellent. To our knowledge, there have been no reports of bioactive glass-associated complications. This corresponds to our experiences. Therefore, we consider the tolerability of BAG to be excellent. This statement in our discussion section does not refer to the patient's outcome (recurrence rate, failure of bone healing, amputation rate). In contrast to the excellent tolerability of BAG, our – unpublished – individual experiences with an absorbable gentamicin-loaded calcium sulfate/hydroxyapatite biocomposite were different. Its use resulted in long-lasting drainage of white fluid like the liquefied bone substitute in most of our patients. This phenomenon of increased drainage of white fluid is also described by other authors using this bone substitute (Drampalos et al., 2019; Pesch et al., 2020). Lastly, Martin McNally concludes that serial debridement followed by defect filling without local antibiotics is not an effective treatment. We discussed the one-stage versus two-stage procedure in our *JBJI* paper. In fact, several authors highlight the possibility of the one-stage use of BAG as well as of other bone graft substitutes.

However, our standard was a two-stage procedure during the inclusion period of the *JBJI* study (July 2013 to January 2017). In addition, our influence of the increased number of previous operations is limited. Most of our patients had already been treated in other hospitals before, and many of the previous operations were required due to large soft tissue defects.

## Data Availability

The data sets analyzed during the current study are available from the corresponding author on reasonable request.
